# Component and Content of Lipid Classes and Phospholipid Molecular Species of Eggs and Body of the Vietnamese Sea Urchin *Tripneustes gratilla*

**DOI:** 10.3390/molecules28093721

**Published:** 2023-04-25

**Authors:** Thi-Kim-Hoa Dinh, Phi-Hung Nguyen, Doan Lan Phuong, Thi-Phuong-Ly Dang, Pham Minh Quan, Thi-Kim-Dung Dao, Valeria P. Grigorchuk, Pham Quoc Long

**Affiliations:** 1 Institute of Natural Products Chemistry, Vietnam Academy of Science and Technology (VAST), 18 Hoang Quoc Viet, Cau Giay, Hanoi 122100, Vietnam; 2 Department of Chemistry, Graduate University of Science and Technology, Vietnam Academy of Science and Technology (VAST), 18 Hoang Quoc Viet, Cau Giay, Hanoi 122100, Vietnam; 3 College of Agriculture and Forestry, Thai Nguyen University (TUAF), Quyet Thang, Thai Nguyen 24119, Vietnam; 4 Federal Scientific Center of the East Asia Terrestrial Biodiversity (Institute of Biology and Soil Science), Far Eastern Branch, Russian Academy of Sciences, Pr-t 100-let Vladivostoka 159, 690022 Vladivostok, Russia

**Keywords:** lipid class, phospholipids, *Tripneustes gratilla*, sea urchin, Cau gai vang

## Abstract

Sea urchins (*Tripneustes gratilla*) are among the most highly prized seafood products in Vietnam because of their nutritional value and medicinal properties. In this research, lipid classes and the phospholipid (PL) molecular species compositions from the body and eggs of *T. gratilla* collected in Hon Tam, Nha Trang, Khanh Hoa, Vietnam, were investigated. Hydrocarbon and wax (HW), triacylglycerol (TG), mono- and diacylglycerol (MDAG), free fatty acid (FFA), sterol (ST), polar lipid (PoL), and monoalkyl-diacylglycerol are the major lipid classes. In PL, five main glycerophospholipid classes have been identified, in which 137 PL molecular species were detected in the body and eggs of *T. gratilla*, including 20 inositol glycerophospholipids (PI), 11 serine glycerophospholipids (PS), 22 ethanolamine glycerophospholipids (PE), 11 phosphatidic acids (PA), and 73 choline glycerophospholipids (PC). PI 18:0/20:4, PS 20:1/20:1, PE 18:1e/20:4, PA 20:1/20:1, and PC 18:0e/20:4 are the most abundant species with the highest content values of 38.65–48.19%, 42.48–44.41%, 41.21–40.03%, 52.42–52.60%, and 7.77–7.18% in each class of the body–eggs, respectively. Interestingly, PL molecules predominant in the body sample were also found in the egg sample. The molecular species with the highest content account for more than 40% of the total species in each molecular class. However, in the PC class containing 73 molecular species, the highest content species amounted to only 7.77%. For both the body and egg TL samples of the sea urchin *T. gratilla*, a substantial portion of C20:4n polyunsaturated fatty acid was found in PI, PE, and PC, but C16, C18, C20, and C22 saturated fatty acids were reported at low levels. The most dominant polyunsaturated fatty acid in PI, PE, and PC was tetracosapolyenoic C20, while unsaturated fatty acid C20:1 was the most dominant in PS and PA. To our knowledge, this is the first time that the chemical properties of TL and phospholipid molecular species of the PoL of Vietnamese sea urchin (*T. gratilla*) have been studied.

## 1. Introduction

The study of marine invertebrates has recently increased, resulting in a rapid increase in the number of publications on lipidomics (fatty acids and lipid classes). However, studies on the molecular species of these invertebrates are limited, especially those within Echinodermata such as sea urchins [1]. Approximately 950 species of sea urchins have been identified globally. Some are considered valuable food sources because their nutritional value and medicinal properties make them commercially, economically, and ecologically important [2].

Sea urchins are a rich source of metabolites, such as lipids, proteins, polypeptides, polysaccharides, carotenoids, vitamins, and minerals. Steroids, saponins, and cerebrosides are secondary but important metabolites of echinoderms. The medicinal and nutritional values of sea urchins are useful for improving the condition and incidence of heart disease. Other health benefits of sea urchins include the prevention of high blood pressure, inflammation, arrhythmia, and cancer [3]. The compositions of lipid classes and fatty acids in sea urchins are attracting many research groups because of their abundance of active lipids, especially omega-3, -6, and -9, in addition to polyunsaturated fatty acids (PUFAs) and essential amino acids [4].

Despite the diversity of data on fatty acids and lipid classes, the number of studies on the molecular species of these invertebrates is still limited, especially on *T. gratilla* [5]. For example, the chemical properties of TL (lipid classes, fatty acid content) and the PoL composition, particularly the glycerophospholipid molecular species in the eggs and body of the Vietnamese sea urchin (*T. gratilla*), are understudied.

## 2. Results and Discussion

### 2.1. Total Lipid, Lipid Classes, and Fatty Acids Composition of T. gratilla

The total lipid (TL) of eggs and body wet weight of *T. gratilla* was identified as 4.41 ± 0.03% and 1.32 ± 0.03%, respectively. The total lipid analysis of the eggs and body samples has resulted in the identification of hydrocarbon and wax (HW), monoalkyl-diacylglycerol (MDAG), triglyceride (TG), fatty acids (FFA), sterol (ST), mono- and diacylglycerol (MADAG), and polar lipid (PoL). Of these, TG was the highest with 78.37 ± 0.64% and 76.10 ± 0.57%, respectively. FA was presented as 4.76 ± 0.03% in the eggs and 4.49 ± 0.03% in the body, while PoLs were 4.41 ± 0.05% and 6.36 ± 0.04%, respectively. The other non-polar lipid classes, including HW, MDAG, and MADAG were less abundant, ranging from 1.11% to 3.31% of the TL [6].

The composition and content of fatty acids (FAs) in the TL of the eggs and body of *T. gratilla* were also characterized (Table 1). As a result, twenty-five and twenty-four fatty acids were observed in the eggs and body samples, respectively, comprising carbon atoms from 12 to 22. It was found that the contents of SFA and MUFA in the eggs were higher than those in the body with 41.74% vs. 17.22% and 26.53% vs. 18.01%, respectively. However, the PUFA content of the eggs alone was 31.08%, about two times less than that of the body (60.50%). The contents of omega-3 (13.97%) and omega-6 (16.79%) in the eggs were lower than those in the body (consisting of 20.67% and 39.83%, respectively), while omega-9 content of the eggs (20.55%) was higher than those in the body (14.73%). The PUFA/SFA ratio of the eggs (0.74) was low compared to the body (3.51), while the n3/n6 ratio of both materials was nearly equal (0.83 vs. 0.52).

Pozharitskaya et al. reported that myristic (14:0) and palmitic (16:0) acids were two major saturated fatty acids (SFA) and eicosenoic acid (20:1ω-9) was a major monounsaturated fatty acid (MUFA). Eicosapentaenoic (20:5ω-3; EPA) acid appeared as the most abundant polyunsaturated fatty acid (PUFA) [7]. In our results, myristic (14:0) and palmitic (16:0) were also two major SFA with 14.50% and 25.10% in the eggs, and 3.59% and 11.74% in the body sample, respectively (Table 1). Eicosenoic acid (20:1ω-9) was not the major MUFA in *T. gratilla*, but (16:1ω-9) and (18:1ω-9) were two major components. Eicosapentaenoic acid (20:5ω-3) reached 6.42% and 13.39%, and (20:4ω-6) reached 10.95% and 30.96% in eggs and body samples, respectively. These were two major polyunsaturated fatty acids (PUFA) in *T. gratilla*. The amounts of MUFA and SFA in body sample were equal at 18.01% and 17.22%, respectively, but there was a significant difference between MUFA (26.53%) and SFA (41.74%) contents in the eggs sample. The total amount of MUFA and SFA was 68.27% in eggs and reached only 35.23% in the body sample.

### 2.2. Polar Lipid Type and Phospholipid Classes

**Figure 1:**
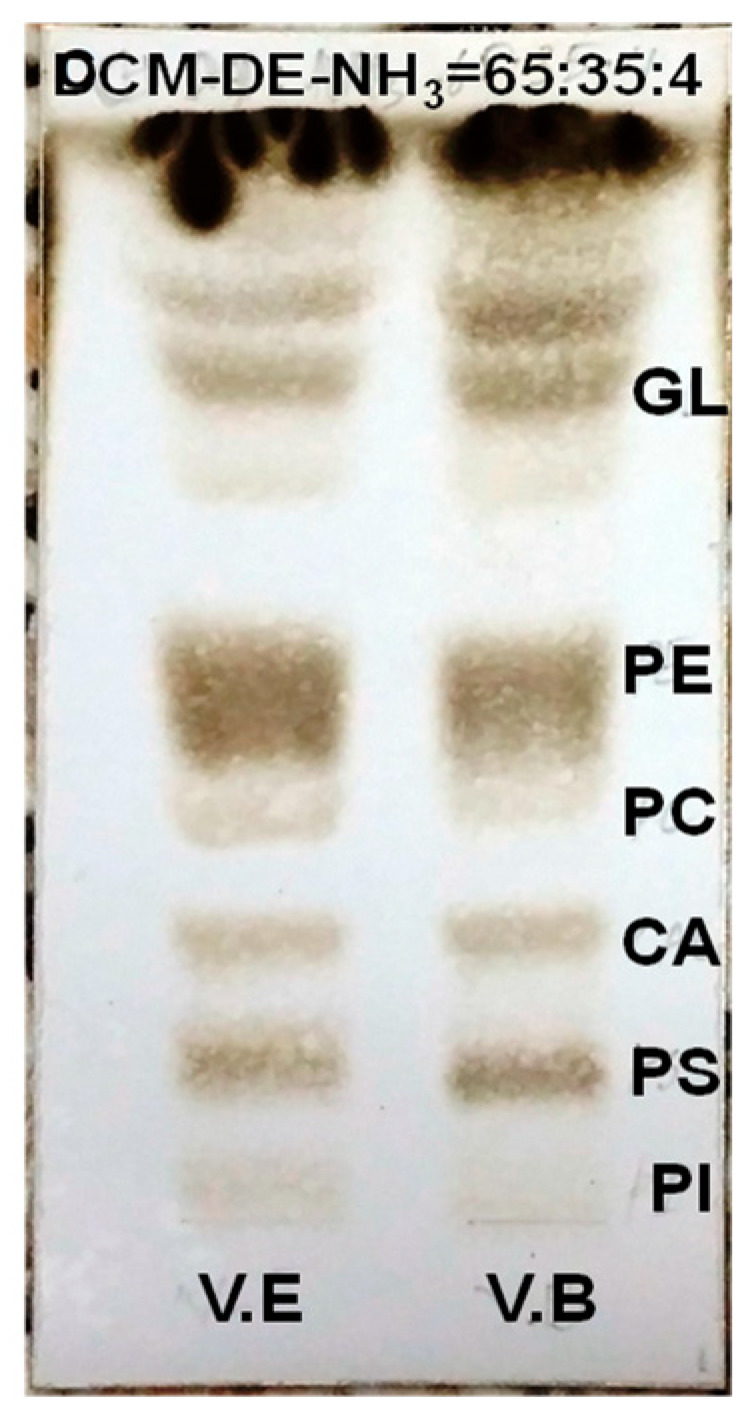
TLC profile analyzing the PoL classes of the eggs and body from *T. gratilla*.

**Figure 2:**
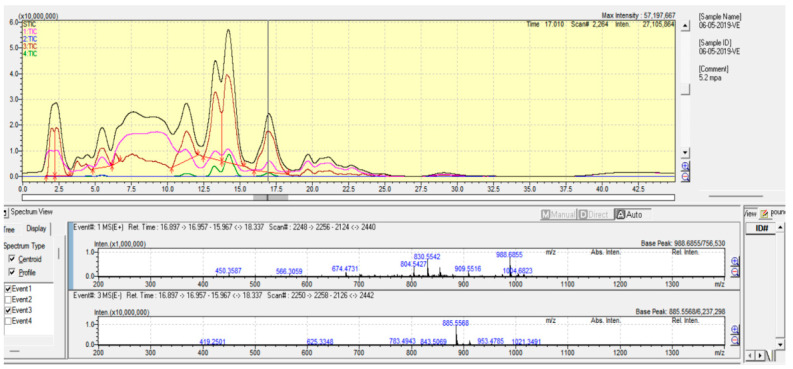
HPLC-HR/MS chromatography of the total PL from the eggs of *T. gratilla*.

In marine invertebrates, the PoL usually consists of glycolipids (GLs) and glycerophospholipids (GPLs) with GPL as the largest PL class. In our study, molecular species of PL from the eggs and body of *T. gratilla* were detected following the previously described HRMS fragmentations of PL standards [8]. Five types of GPLs, including phosphatidylinositol (PI), phosphatidylserine (PS), phosphatidylethanolamine (PE), phosphatidic acids (PA), and phosphatidylcholine (PC), were identified (Figure 1 and Figure 2). Their molecular structures and contents were analyzed using a Shimadzu LCMS-IT-TOF instrument with a Shimadzu LCMS Solution control and processing software (v.3.60.361, Shimadzu, Kyoto, Japan).

The polar ends of the structures of the identified GPLs classes, inositol, serine, ethanolamine, and choline were major groups in the polar head contained in their molecules.

### 2.3. Molecular Species of Phosphatidylinositol (PI)

In total, 20 molecular species were found in the phosphatidylinositol (PI) class from the PoL in both samples of the sea urchin *T. gratilla* (Table 2). Among these, PI 18:0/20:4 (*m/z* [M − H]^−^ 885.5562) was the highest peak with 38.65% (eggs) and 48.19% (body) content in the total PI species. PI 20:0/20:5 (*m*/*z* [M − H]^−^ 911.5645, calc. for C_49_H_85_O_13_P), PI 20:0/20:4 (*m/z* [M − H]^−^ 913.5814, calc. for C_49_H_87_O_13_P), and PI 18:0/20:5 (*m/z* [M − H]^−^ 883.5401, calc. for C_47_H_81_O_13_P) followed by 13.37%, 11.49%, and 9.36% content in the eggs and 12.64%, 9.89%, and 6.96% content in the body, respectively. PI 16:0/20:4, PI 18:0e/20:4, PI 18:0/20:3, and PI 20:1/20:5 were in the third highest group with the contents ranging from 2.17% to 4.82%. The other species presented low contents that were less than 1% (see Table 2 for detail).

**Figure 3:**
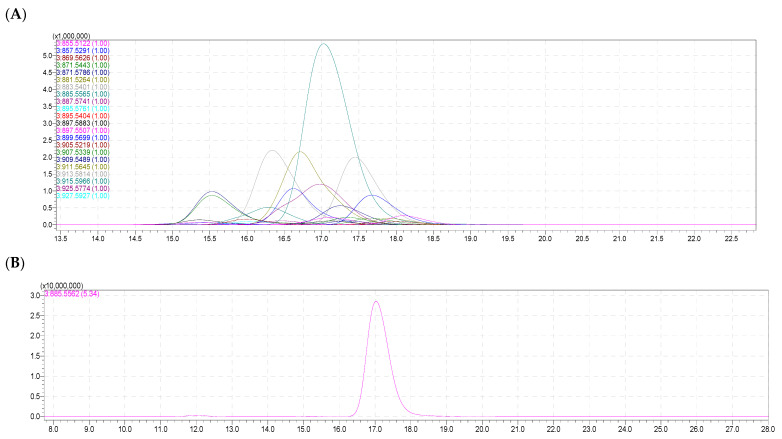

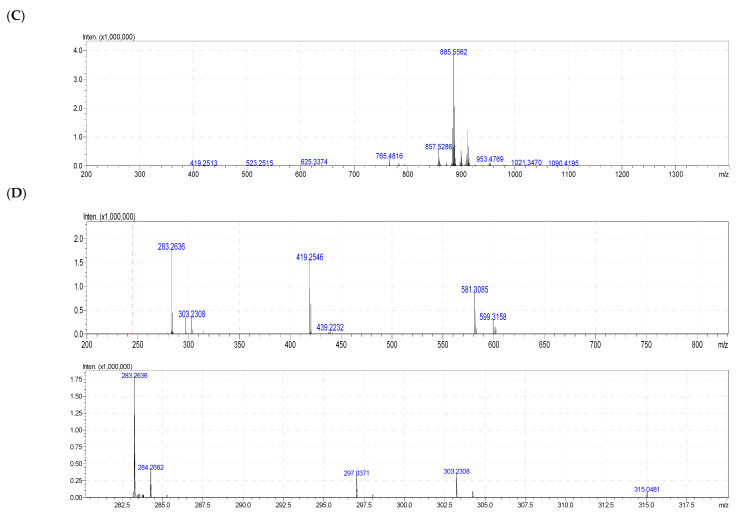
(**A**) The HPLC–HR/MS of total molecular species of the PI class. (**B**) HPLC–HR/MS of the molecular species PI at *m/z* 885.5662. (**C**) Fragmentation MS^−^ and (**D**) MS^2−^ of PI 18:0/20:4.

The structure of PI can be characterized according to their MS^–^ and MS/MS^–^ data. Signals of negative quasi-molecular ions [M – H]^–^ were observed in the HRMS spectra of all components of the formula species of PI (Figure 3A and Table 2).

For example, a negative quasi-molecular ion [M – H]^−^ at *m/z* 885.5562 for PI 18:0/20:4 was detected and assigned for a molecular formula of C_47_H_83_O_13_P with a calculated value of 885.5571 and a different value of 0.0009 (Figure 3B,C). This was the strongest signal (highest peak) in the HPLC–HR/MS of total molecular species of the PI class, with a retention time (Rt) of 17.939 min (Figure 3A,B). From the MS^2−^ spectrum of the ions [M – H]^−^ of this PI 38:4 (Figure 3D), one signal corresponding to one carboxylate anion of 20:4 was detected at *m/z* 303.2308 (calc. for C_20_H_32_O_2_).

**Figure 4:**
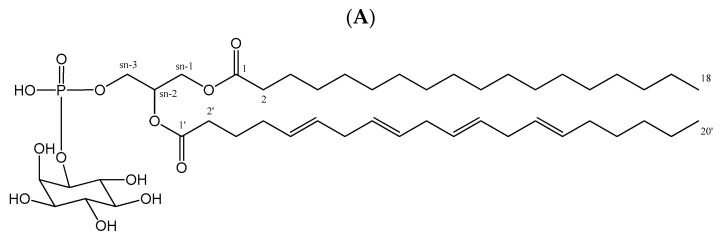

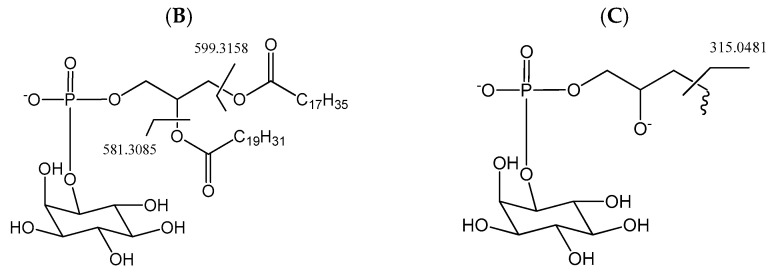
(**A**) Chemical structure of the identified molecular species PI 18:0/20:4. (**B**,**C**) Fragmentations of PI 38:4 with MS^2−^ assignment.

Furthermore, the above observation was supported by a peak appearing at *m/z* 581.3085 which was calculated for C_27_H_50_O_11_P^−^ (Figure 4B). For the structure of PI, the fatty acid (FA) anion ([RCOO]–) was liberated from the *sn*^−1^ position due to the alkenyl linkages at the *sn*^−2^ position (Figure 4). Thus, the peak that appeared at *m/z* 283.2636 could be assigned for the loss of the fatty acid (C18:0) anion (C_18_H_35_O_2_^−^) at *sn*^−1^ in the molecular species of PI 38:4, and this observation was also supported by a signal corresponding to C_29_H_46_O_11_P^−^ at *m/z* 599.316 (Figure 3D and Figure 4B).

The ion peak at *m*/*z* 315.0481 corresponded to a fragmentation of a partial structure glycerol phosphatidylinositol unit (Figure 4C), which could mean the loss of two fatty acid units (C18:0) and (C20:4) at *sn*^−1^ and *sn*^−2^ in the molecular species PI 38:4. From the observation above, the molecular species of PI 38:4 was therefore characterized as diacylglycerol phosphatidylinositol 18:0/20:4, and its structure is presented in Figure 4A.

### 2.4. Molecular Species of Phosphatidylserine (PS)

**Figure 5:**
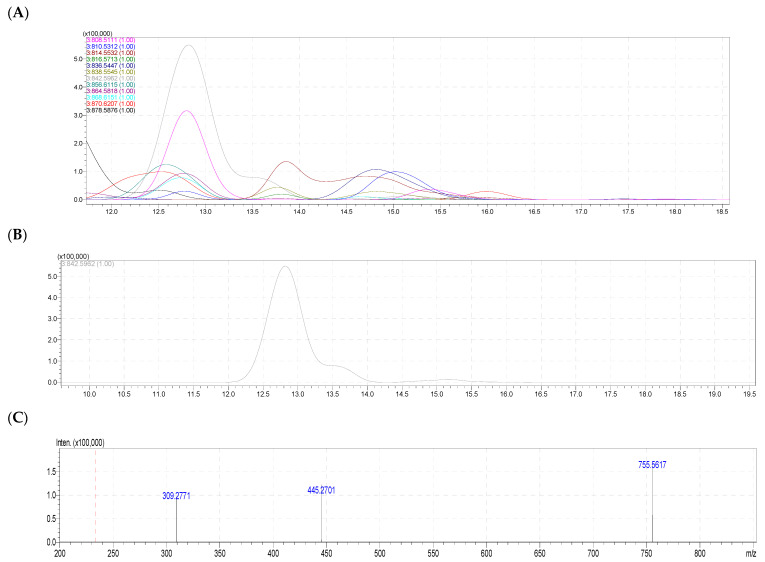

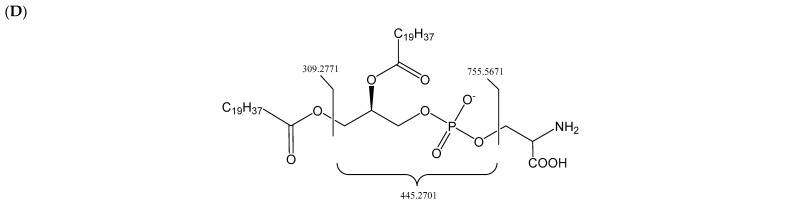
(**A**) The HPLC–HR/MS of total molecular species of PS class. (**B**) HPLC–HR/MS of the molecular species PS at *m*/*z* 842.5962. (**C**) Fragmentation MS^2–^ of PS 20:1/20:1. (**D**) Fragmentations of PS 40:2 (PS 20:1/20:1) with MS^2–^ assignment.

In the phosphatidylserine (PS) class from the PL of both samples of the Vietnamese sea urchin *T. Gratilla*, a total of 11 PS molecular species have been identified (Figure 5 and Table 3).

Of these, PS 20:1/20:1 with quasi-molecular ions [M – H]^−^ at *m/z* 842.5962 (deduced for an MF C_46_H_86_NO_10_P) was the highest with 42.48% content in the egg and 44.41% content in the body (Figure 5). PS 20:1/21:1 (*m/z* [M – H]^−^ 856.6115, calc. for C_47_H_88_NO_10_P) and PS 20:1/22:1 (*m/z* 870.6207, calc. for C_48_H_90_NO_10_P) followed next with 10.43% and 7.31% in the eggs and 14.60% and 11.32% in the body, respectively. Except for PS 20:1/20:4 with a content of up to 9.67% in the eggs, the other PS molecular species presented the contents that were under 9%. There were two species, PS 38:5 and PS 20:1/18:1, which presented only 0.50% and 0.60% in body sample, respectively (see Table 3 for detail).

### 2.5. Molecular Species of Phosphatidylethanolamine (PE)

**Figure 6:**
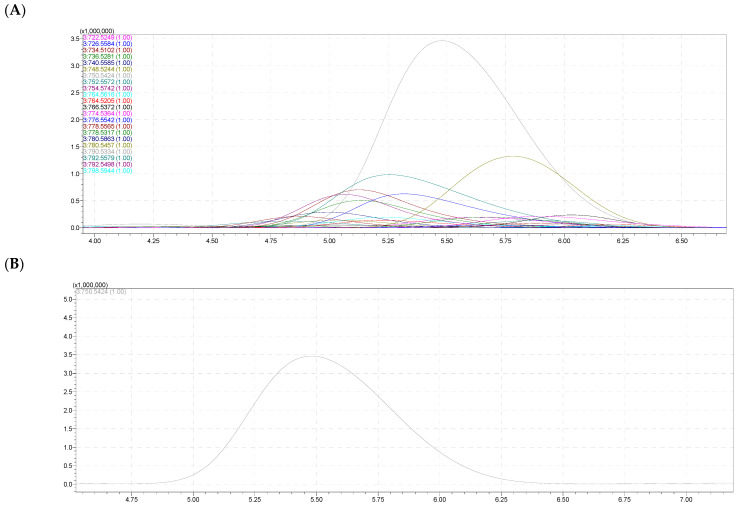

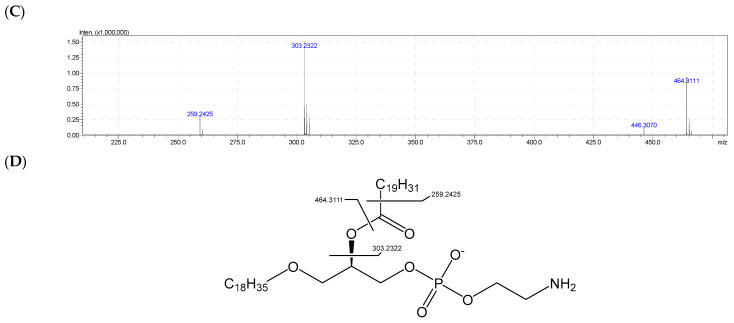
(**A**) The HPLC–HR/MS of total molecular species of PE class. (**B**) HPLC–HR/MS of a molecular species PE at *m*/*z* 750,5424. (**C**) Fragmentation MS^2−^ of PE 18:1e/20:4. (**D**) Fragmentations of PE 38:5e (PE 18:1e/20:4) with MS^2−^ assignment.

For phosphatidylethanolamine (PE), 22 molecular species were found (Table 4 and Figure 6A). Among these 22 components, 3 presented high content (over 10%) including PE 18:1e/20:5, PE 18:1e/20:4, and PE18:1e/20:2, accounting for 59.75% in the eggs and 60.31% in the body of total PE species. The remaining species presented low contents that were under 10%. There was no variation in the identified molecular species and little variation in the content of each composition between the two samples (eggs and body). In this study, the letter “e” denotes the head attached to the glycerol molecule with an ether bond.

Among the received signals of the PE class, the negative ion signal [M – H]^−^ had the highest intensity at *m/z* 750.5424 in both the eggs and body samples (41.21% and 40.03%), and the signal corresponding to the PE molecular species occupied the highest concentration in this class. The calculated molecular formula was C_43_H_78_NO_7_P which had seven oxygen atoms in the molecule, defining an acyl-alkyl PE. On the negative ion spectrum MS^2−^ of ion [M – H]^−^ 750.5424, there were signals at *m/z* 303.2322 corresponding to the fatty acid anion C_20_H_32_O_2_ (C_19_H_31_COOH, 20:4n); the signal at *m/z* 259.2425 corresponded to the anion C_19_H_32_; the signal at *m/z* 464.3111 corresponded to the molecular ion losing a neutral fragment C_20_H_30_O (C_19_H_31_COOH–H_2_O); and the signal at *m/z* 446.3070 corresponded to the molecular ion losing a neutral fragment, the fatty acid C_20_H_32_O_2_ (Figure 6C,D).

### 2.6. Molecular Species of Phosphatidic Acids (PA)

**Figure 7:**
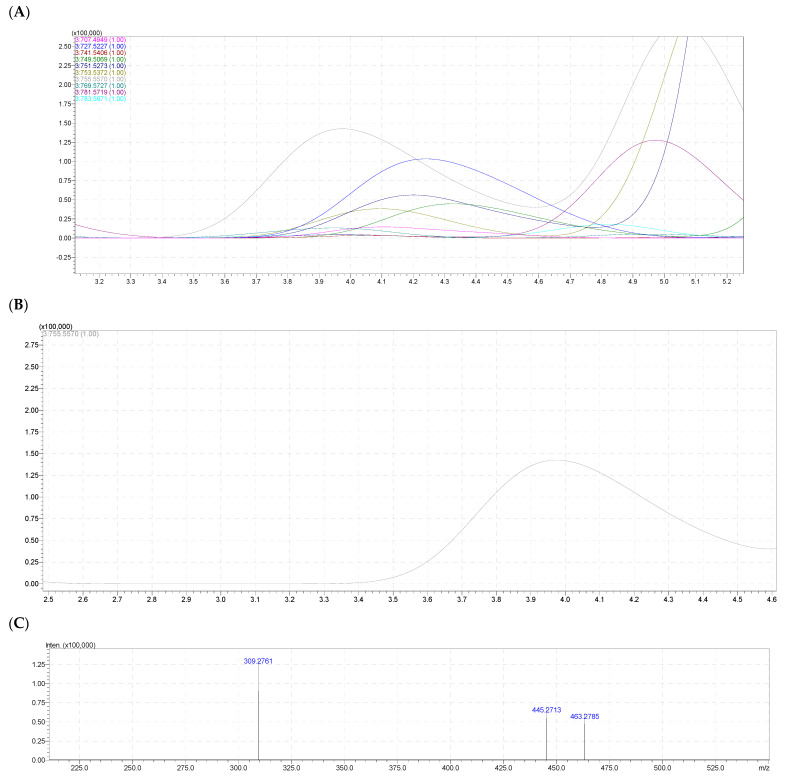

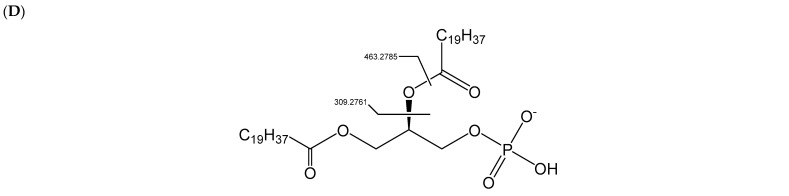
(**A**) The HPLC–HR/MS of total molecular species of PA class. (**B**) HPLC–HR/MS of the molecular species PA at *m/z* 755,5570. (**C**) Fragmentation MS^2−^ of PA 20:1/20:1. (**D**) Fragmentations of PA 40:2 (PA 20:1/20:1) with MS^2−^ assignment.

In the phosphatidic acid PA class, 11 molecular forms were identified (Table 5 and Figure 7). Among the received signals, the negative ion signal [M – H]^–^ had the highest intensity at *m/z* 755.5570 in both the eggs and body TL samples of sea urchin *T. gratilla*, and the signal corresponding to the molecular species occupied the highest concentration in this PA class (Figure 7A,B).

The calculated molecular formula was C_43_H_81_O_8_P which consisted of eight oxygen atoms in the molecule, defining a diacyl PA. On the negative ion spectrum MS^2–^ of ion [M – H]^–^ 755.5570, it could be seen that signals at *m/z* 309.2761 belonged to the anion of fatty acid C_20_H_38_O_2_ (20:1n); the signal at *m/z* 463.2785 corresponded to the molecular ion that lost a neutral fragment C_20_H_36_O (C_19_H_37_COOH–H_2_O); and the signal at *m/z* 445.2713 corresponds to the molecular ion losing a neutral fragment, the fatty acid C_20_H_38_O_2_ (Figure 7C).

### 2.7. Molecular Species of Phosphatidylcholine (PC)

**Figure 8:**
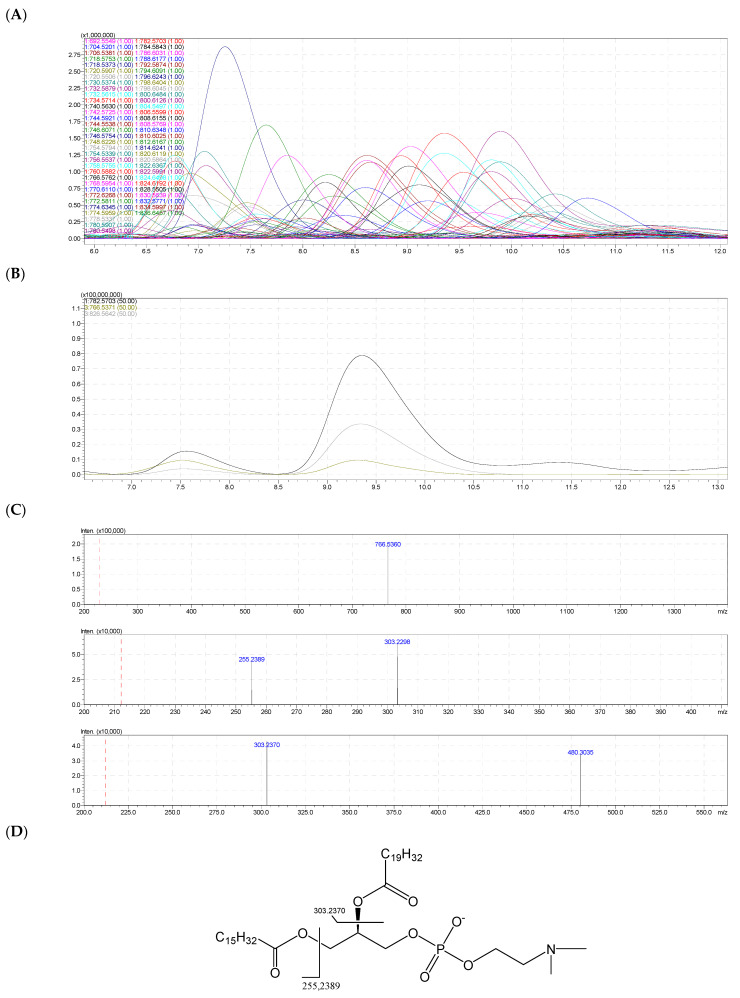
(**A**) The HPLC–HR/MS of total molecular species of PS class. (**B**) HPLC–HR/MS of the molecular species PC at *m/z* 782.5703. (**C**) Fragmentation MS^2−^ of PC 16:0/20:4. (**D**) Fragmentations of PC 36:4 (PC 16:0/20:4) with MS^2−^ assignment.

From the two samples (eggs and body) of sea urchin *T. gratilla*, 73 signals were identified in the composition of the phosphatidylcholine (PC) class (Table 6), corresponding to 73 molecular species (Table 5 and Figure 8A). Among them, the positive molecular ion signals [M + H]^+^ at *m/z* 782.5703 had the highest intensity. The signals were observed simultaneously on the negative ion spectra of the [M + CH_3_COO]^−^ and [M − CH_3_]^−^ ions at *m/z* 826.5642 and 766.5371 (Figure 8B). The molecular formula was determined as C_44_H_80_NO_8_P which had eight oxygen atoms in the molecule structure, defining a diacyl PC.

On the negative ion spectrum -MS^2^ of the ion [M + CH_3_COO]^−^ 826.5642, there was a signal at *m/z* 766.5360 corresponding to the anion [M + CH_3_COO − C_3_H_6_O_2_]^−^ (Figure 8C).

The MS^2^ spectrum of the ion with *m/z* 766.5371 showed fragment ions as follows: the ion with a signal at *m/z* 255.2389 corresponded to the fatty acid anion C_16_H_32_O_2_ (16:0); the ion with a signal at *m/z* 303.2370 corresponded to the anion C_20_H_32_O_2_ (20:4n); the ion with a signal at 480.3035 matched with a molecular ion losing a neutral fragment C_20_H_30_O (C_19_H_31_COO–H_2_O) (see Figure 8D). In addition, the chemical structure of the PC 16:0/20:4 was consistent with the other data. Therefore, the identified PC molecular species was identified as PC 16:0/20:4.

This study examined the lipid composition and content of sea urchins (*T. gratilla*) by comparing the extracts taken from eggs and body samples. Lipid classes were identified with the TLC method and the total lipid content was analyzed using Sorbfil TLC Video densitometer (Krasnodar, Russia) software. Furthermore, the non-polar lipid and polar lipid (PoL) were separated via chromatography. Fatty acid (FA) content and compositions were analyzed via GC-MS. This research advances the study of content and identification of polar lipid classes by using LC-MS/MS technique. As a result, five types of phospholipids were identified: PI, PS, PE, PA, and PC with 137 molecular species in total for each sample. Among those, PI 18:0/20:4; PS 20:1/20:1; PE 18:1e/20:4, and PC 16:0/20:4 were the most abundant phospholipid species with the highest contents of 38.65–48.19%, 42.48–44.41%, 41.21–40.03%, 52.42–52.60%, and 7.77–7.18% in each class of the body–eggs, respectively. Our data was the first to show phospholipid molecular species of PoL in the Vietnamese sea urchin (*T. gratilla*).

Imbs et al. reported that TG, WE, and DAGE were major classes of non-polar lipids in marine invertebrates [1], while the major polar lipid classes were phospholipids containing PC, PE, PS, and PI as glycerophospholipids (GPL). This study found that TG was the highest class with around 76–78%, but HW and MADAG were the two lowest classes with percentages between 1.1% and 2.3% for both eggs and body, respectively. Moreover, Kostetsky et al. reported the composition of PC and PE molecular species in a Japanese sea urchin *Strongylocentrotus intermedius* with alkylacyl PC and alkenylacyl PE were the dominant forms [5]. In detail, 29 PE and 26 PC molecular species were structurally identified. In addition, Imbs et al. also showed that the major PC molecular species were 18:1a/20:5, 16:0a/20:5, 18:0a/20:5, 18:1/20:5, 18:0/20:5, 20:1/20:5, and 16:0/20:5 and the major PE molecules were 18:0p/20:4, 18:0p/20:5, 18:0a/20:5, 18:0/20:4, 18:0/20:5, 18:1/20:5, and 20:1/20:5 in the muscle tissues of echinoderms. Our results showed the identification of five types of phospholipids including PI, PS, PE, PA, and PC with 137 molecular species in total for each sample with PI, PE, and PC being major classes. Among these, twenty PI molecular species, twenty-two PE molecular species, and seventy-three PC molecular species were structurally identified. It was also found that PI 18:0/20:4, PE 18:1e/20:4, and PC 16:0/20:4 were the most abundant phospholipid molecular species.

## 3. Materials and Methods

### 3.1. Material

A wild-caught sample of Cau gai vang *T. gratilla* (Linnaeus, 1758) was collected by divers in shallow water using specialized tools from the intertidal zone to a depth of about 70 m, on 17 November 2016, from Hon Tam Island, Nha Trang, Khanh Hoa, Vietnam (12°10′33.3″ N, 109°14′34.8″ E). Its scientific name was examined by Dr. Nguyen An Khang, Nha Trang Institute of Oceanography, Vietnam Academy of Science and Technology. After collection, the samples were stored in an insulated container and kept at a temperature of 0 to 4 °C while being transported by airplane to the laboratory in Hanoi. The total time of shipment was 2.5 h. A voucher specimen was deposited at the Institute of Natural Products Chemistry, VAST.

### 3.2. Extraction of Total Lipid

The sea urchins were cut in half and washed with distilled water. Eggs were separated from the body using a spoon. The eggs (TG-E) and body (TG-B) materials were immediately homogenized with a blender under cool conditions for 2 min before extracting the total lipid by using the Bligh and Dyer method [9]. Briefly, the eggs and body (each 300 g) materials were extracted with 900 mL of CHCl_3_–MeOH (*v*/*v* = 1/2), the solid:liquid ratio being 1:3 (g/mL), and then sonicated for 2 h. Afterward, 300 mL of CHCl_3_ and 600 mL of distilled water were added to the mixture for partitioning it. When partitioned, the lower layer (containing lipid) was separated, and the residue (upper layer) was extracted twice using continuous sonication for 2 h. Thus, the total extraction time was 6 h for one sample and the procedure was repeated three times. The combined lipid extract solution was dehydrated via anhydrous Na_2_SO_4_ and was evaporated to give a total lipid extract. The total lipid content was calculated as a percentage of lipid quantity compared to the fresh sample weight. Finally, the lipid extract was stored in pure CHCl_3_ at −20 °C. Lipid samples used for further analyses were prepared daily by diluting them in a mixture of CHCl_3_ and MeOH (*v*/*v* = 1:2).

### 3.3. Analysis of Polar Lipid Classes

Polar lipid classes composition was first analyzed via thin-layer chromatography (TLC) using silica gel plates (Sorbfil, Krasnodar, Russia) and a solvent system of dichloromethane/diethyl ether/NH_3_ (65:35:4, *v*/*v*/*v*). Then, the TLC plate was sprayed with 10% H_2_SO_4_ in MeOH and heated at 240 °C for 10 min after air-drying. The PL classes were detected by comparing their Rf values with each standard sample. Polar lipid standards PC (16:0–20:4), PE (16:0–20:4), PS (16:0–20:4), PI (18:0–20:4), C18(Plasm)-20:4 PC, and C18(Plasm)-20:4 PE were purchased from Avanti Polar Lipids Co. (Alabaster, AL, USA). 

### 3.4. Analysis of Molecular Species of Phospholipids

The molecular species of polar lipids (phospholipids) from eggs and body materials were detected using previously described HRMS fragmentations of PL standards [10,11]. The high-performance liquid chromatography/high-resolution tandem ion trap–time of the flight mass spectrometry with a Shimadzu LCMS-IT-TOF instrument (Shimadzu, Kyoto, Japan) was used to analyze the molecular species of PL. The LCMS-IT-TOF equipped with two LC-20AD pump units, a high-pressure gradient forming module, 9CCTO-20A column oven, SIL-20A auto sampler, CBM-20A communications bus module, DGU-20A3 degasser, and a Shim-Pack diol column (50 mm × 4.6 mm ID, 5 μm particle size) was operated both at positive and negative ion modes during each analysis at electrospray ionization (ESI) conditions. The ion source temperature was 200 °C, the range of detection was *m*/*z* 200–1600, and the potential in the ion source was −3.5 and 4.5 kV for negative and positive modes, respectively. The drying gas (N2) pressure was 200 kPa. The nebulizer gas (N2) flow was 1.5 L/min. The mobile phase condition for separation of PoL was performed using a binary gradient consisting of solvent mixture A: *n*-hexane/2-propanol/acid formic/(C_2_H_5_)_3_N (82:17:1:0.08, *v*/*v*/*v*/*v*) and mixture B: 2-propanol/H_2_O/acid formic/(C_2_H_5_)_3_N (85:14:1:0.08, *v*/*v*/*v*/*v*). The gradient started at 5% of mixture B, and its percentage increased to 80% over 25 min. This composition was maintained for 1 min before being returned to 5% of mixture B for more than 10 min and maintained at 5% for another 4 min (total run time of 40 min). The flow rate was 0.2 mL/min. Polar lipids were detected via high-resolution mass spectrometry (HRMS) and compared against authentic standards using a Shimadzu LCMS Solution control and processing software (v.3.60.361, Shimadzu, Kyoto, Japan). Individual molecular species within each PL class was measured by calculating the peak areas on extracted ion chromatograms [12].

## 4. Conclusions

Through experimentation to determine the total lipid and lipid classes of both eggs and body material from sea urchin *T. Gratilla*, this study found that the total lipid of eggs was much higher than that of the body sample (4.41% vs. 1.32% on wet weight base, respectively). The seven lipid classes of total lipid were hydrocarbon and wax (HW), triacylglycerol (TAG), free fatty acids (FFA), sterol (ST), polar lipid (PoL), and monoalkyl-diacylglycerol (MADAG). Among these, the proportions of TAG accounted for the highest amount from approximately 76% to 78%. The two lowest classes were HW and MADAG with percentages of 1.1% and 2.3% of the total lipid for eggs and body materials, respectively.

To our knowledge, this is the first study to determine the total lipid, lipid classes, fatty acid compositions, and phospholipid molecular species of sea urchins in general and *T. gratilla* in particular. The five types of phospholipids were identified as PI, PS, PE, PA, and PC with 137 molecular species in total for each sample. PI 18:0/20:4, PS 20:1/20:1, PE 18:1e/20:4, and PC 16:0/20:4 were the most abundant phospholipid species with the highest contents of 38.65–48.19%, 42.48–44.41%, 41.21–40.03%, 52.42–52.60% and 7.77–7.18% in each class of the body–eggs, respectively.

In both body and eggs, PoLs of the sea urchin *T. gratilla*, C20:4n was the most abundant polyunsaturated fatty acid in PI, PE, and PC classes, while C16, C18, C20, and C22 saturated fatty acids were less common. The most dominant polyunsaturated fatty acid in PI, PE, and PC was tetracosapolyenoic C20, while unsaturated fatty acid C20:1 was the most dominant in PS and PA classes. To our knowledge, this is the first time that the chemical properties of TL, especially the phospholipid molecular species of the PoL in the Vietnamese sea urchin (*T. gratilla*), have been studied.

## Figures and Tables

**Table 1:** Composition and content of fatty acids in the eggs and body samples of the Vietnamese sea urchin *T. gratilla*.

No.	Fatty Acids	Eggs of *T. gratilla* (%)	Body of *T. gratilla* (%)	No.	Fatty Acids	Eggs of *T. gratilla* (%)	Body of *T. gratilla* (%)
1	12:0	0.08	-	15	18:0	1.39	1.57
2	14:0	14.50	3.59	16	20:0	0.23	0.12
3	14:1 (n-7)	2.03	0.33	17	20:3 (n-3)	0.32	0.68
4	15:0	0.44	0.20	18	20:2 (n-6)	0.67	-
5	16:1 (n-9)	8.66	3.59	19	20:1 (n-9)	2.50	6.25
6	16:2 (n-4)	0.32	-	20	20:4 (n-6)	10.95	30.96
7	16:1 (n-7)	3.08	2.04	21	20:5 (n-3)	6.42	13.39
8	16:0	25.10	11.74	22	20:3 (n-6)	2.46	5.55
9	18:4 (n-3)	3.67	2.64	23	20:4 (n-3)	1.15	1.46
10	18:2 (n-6)	1.86	1.81	24	22:6 (n-3)	0.22	0.31
11	18:1 (n-9)	8.87	4.62	25	22:1 (n-9)	0.55	0.27
12	18:1 (n-7)	0.87	0.91	26	22:6 (n-6)	-	0.30
13	18:3 (n-3)	2.19	2.19	27	22:4 (n-6)	-	0.57
14	18:3 (n-6)	0.85	0.64	28	others	0.65	4.27
SFA		41.74	17.22	Omega-6		16.79	39.83
MUFA		26.53	18.01	Omega-9		20.55	14.73
PUFA		31.08	60.50	PUFA/SFA		0.74	3.51
Omega-3		13.97	20.67	n3/n6		0.83	0.52

**Table 2:** Molecular species of phosphatidylinositol (PI) identified from the PL of the egg and body of the Vietnamese sea urchin *T. gratilla*.

No.	Molecular Species	Molecular Weight [M − H]^−^	Molecular Formula (MF)	Retention Time (Rt, min)	Content in Total PI (%)	
					Egg	Body
1.	PI 16:0/20:5	855.5122	C_45_H_77_O_13_P	18.308	0.77	0.07
2.	PI 16:0/20:4	857.5291	C_45_H_79_O_13_P	17.893	3.36	1.44
3.	PI 18:0e/20:5	869.5626	C_47_H_83_O_12_P	16.239	0.69	0.54
4.	PI 17:0/20:4	871.5443	C_46_H_81_O_13_P	17.568	0.90	0.46
5.	PI 18:0e/20:4	871.5786	C_47_H_85_O_12_P	15.748	4.82	4.71
6.	PI 18:1/20:5	881.5264	C_47_H_79_O_13_P	18.156	0.57	0.17
7.	PI 18:0/20:5	883.5401	C_47_H_81_O_13_P	17.661	9.36	6.96
8.	PI 18:0/20:4	885.5562	C_47_H_83_O_13_P	17.212	38.65	48.19
9.	PI 18:0/20:3	887.5741	C_47_H_85_O_13_P	16.938	2.17	2.19
10.	PI 20:1e/20:4	897.5883	C_49_H_87_O_12_P	15.551	0.69	0.40
11.	PI 19:0/20:5	897.5507	C_48_H_83_O_13_P	17.327	1.23	1.08
12.	PI 19:0/20:4	899.5699	C_48_H_85_O_13_P	16.873	5.92	6.43
13.	PI 40:8	905.5219	C_49_H_79_O_13_P	18.195	0.44	0.55
14.	PI 20:3/20:4	907.5339	C_49_H_81_O_13_P	17.870	0.72	0.50
15.	PI 20:1/20:5	909.5489	C_49_H_83_O_13_P	17.444	2.91	2.21
16.	PI 20:0/20:5	911.5645	C_49_H_85_O_13_P	16.959	13.37	12.64
17.	PI 20:0/20:4	913.5814	C_49_H_87_O_13_P	16.573	11.49	9.89
18.	PI 20:0/20:3	915.5966	C_49_H_89_O_13_P	16.273	0.69	0.46
19.	PI 21:1/20:4	925.5774	C_50_H_87_O_13_P	16.684	0.71	0.87
20.	PI 21:0/20:4	927.5927	C_50_H_89_O_13_P	16.243	0.42	0.24

**Table 3:** Molecular species of phosphatidylserine (PS) identified from PoL of the egg and body of the Vietnamese sea urchin *T. gratilla*.

No.	Molecular Species	Molecular Weight [M – H]^−^	Molecular Formula (MF)	Retention Time (Rt, min)	Content in Total PS (%)	
					Eggs	Body
1.	PS 38:5	808.5111	C_44_H_76_NO_10_P	15.524	1.27	0.50
2.	PS 18:0/20:4	810.5312	C_44_H_78_NO_10_P	15.067	5.47	3.88
3.	PS 20:1/18:1	814.5532	C_44_H_82_NO_10_P	13.959	5.87	0.60
4.	PS 20:1/20:4	836.5447	C_46_H_80_NO_10_P	14.747	9.67	5.30
5.	PS 40:4	838.5545	C_46_H_82_NO_10_P	13.907	2.68	1.53
6.	PS 20:1/20:1	842.5962	C_46_H_86_NO_10_P	12.900	42.48	44.41
7.	PS 20:1/21:1	856.6115	C_47_H_88_NO_10_P	12.646	10.43	14.60
8.	PS 20:1/22:4	864.5818	C_48_H_84_NO_10_P	12.874	7.71	6.03
9.	PS 20:1/22:2	868.6151	C_48_H_88_NO_10_P	12.815	5.43	8.09
10.	PS 20:1/22:1	870.6207	C_48_H_90_NO_10_P	12.338	7.31	11.32
11.	PS 21:1/22:4	878.5876	C_49_H_86_NO_10_P	12.629	2.07	2.70

**Table 4:** Molecular species of phosphatidylethanolamine (PE) identified from PoL of the egg and body of the Vietnamese sea urchin *T. gratilla*.

No.	Molecular Species	Molecular Weight [M – H]^−^	Molecular Formula (MF)	Retention Time (Rt, min)	Content in Total PE (%)	
					Egg	Body
1.	PE 16:1e/20:4	722.5249	C_41_H_74_NO_7_P	5.931	2.83	3.58
2.	PE 36:3e	726.5584	C_41_H_78_NO_7_P	5.619	1.15	1.80
3.	PE 37:6e	734.5102	C_42_H_74_NO_7_P	5.986	0.62	0.41
4.	PE 17:1e/20:4	736.5281	C_42_H_76_NO_7_P	5.724	2.11	2.47
5.	PE 37:3e	740.5585	C_42_H_80_NO_7_P	5.250	0.42	0.93
6.	PE 18:1e/20:5	748.5244	C_43_H_76_NO_7_P	5.811	10.56	6.60
7.	PE 18:1e/20:4	750.5424	C_43_H_78_NO_7_P	5.531	41.21	40.03
8.	PE 18:0e/20:4	752.5572	C_43_H_80_NO_7_P	5.365	6.88	5.28
9.	PE18:1e/20:2	754.5742	C_43_H_82_NO_7_P	5.141	7.98	13.68
10.	PE 19:1e/20:4	764.5616	C_44_H_80_NO_7_P	5.304	1.85	1.63
11.	PE 18:1/20:4	764.5205	C_43_H_76_NO_8_P	6.382	1.41	2.95
12.	PE 18:0/20:4	766.5372	C_43_H_78_NO_8_P	6.161	2.52	3.41
13.	PE 20:2e/20:5	774.5364	C_45_H_78_NO_7_P	5.662	1.62	0.97
14.	PE 20:2e/20:4	776.5542	C_45_H_80_NO_7_P	5.381	6.58	4.41
15.	PE 20:1e/20:4	778.5565	C_45_H_82_NO_7_P	5.205	6.56	5.25
16.	PE 39:5	778.5317	C_44_H_78_NO_8_P	6.057	0.31	0.14
17.	PE 20:2e/20:2	780.5863	C_45_H_84_NO_7_P	5.075	2.25	2.34
18.	PE 39:4	780.5457	C_44_H_80_NO_8_P	5.085	0.14	0.11
19.	PE 40:8e	790.5334	C_45_H_78_NO_8_P	6.204	0.53	0.59
20.	PE 20:1/20:4	792.5579	C_45_H_80_NO_8_P	4.945	1.07	1.12
21.	PE 20:1/20:4 isomer	792.5498	C_45_H_80_NO_8_P	5.996	1.44	1.77
22.	PE 20:1/20:1	798.5944	C_45_H_86_NO_8_P	5.237	0.27	0.54

**Table 5:** Molecular species of phosphatidic acids (PA) identified from PoL of the egg and body of the Vietnamese sea urchin *T. gratilla*.

No.	Molecular Species	Molecular Weight [M – H]^−^	Molecular Formula (MF)	Retention Time (Rt, min)	Content in Total PA (%)	
					Egg	Body
1.	PA 18:1e/20:4	707.4949	C_41_H_73_O_7_P	4.131	3.09	2.64
2.	PA 20:1/18:1	727.5227	C_41_H_77_O_8_P	4.162	19.18	1.72
3.	PA 38:1	729.5479	C_41_H_79_O_8_P	2.064	1.07	1.70
4.	PA 39:2	741.5406	C_42_H_79_O_8_P	4.074	2.17	3.43
5.	PA 40:5	749.5069	C_43_H_75_O_8_P	4.329	7.95	6.29
6.	PA 40:4	751.5273	C_43_H_77_O_8_P	4.200	8.76	3.45
7.	PA 40:3	753.5372	C_43_H_79_O_8_P	4.105	6.07	4.84
8.	PA 20:1/20:1	755.5570	C_43_H_81_O_8_P	3.904	52.42	52.60
9.	PA 20:1/21:1	769.5727	C_44_H_83_O_8_P	3.953	6.69	10.75
10.	PA 20:1/22:2	781.5719	C_45_H_81_O_8_P	3.963	3.96	7.06
11.	PA 42:2	783.5871	C_45_H_85_O_8_P	3.915	2.83	5.52

**Table 6:** Molecular species of phosphatidylcholine (PC) identified from PoL of the egg and body of the Vietnamese sea urchin *T. gratilla*.

No.	Molecular Species	Molecular Weight [M + H]^+^	Molecular Formula (MF)	Retention Time (Rt, min)	Content in Total PC (%)	
					Egg	Body
1.	PC 30:0e	692.5549	C_38_H_78_NO_7_P	8.461	0.61	0.85
2.	PC 14:0/16:1	704.5201	C_38_H_74_NO_8_P	10.767	1.05	0.69
3.	PC 31:0e	706.5763	C_39_H_80_NO_7_P	4.094	0.21	0.27
4.	PC 14:0/16:0	706.5381	C_38_H_76_NO_8_P	10.214	1.33	1.95
5.	PC 16:0e/16:1	718.5753	C_40_H_80_NO_7_P	8.261	0.67	1.38
6.	PC 31:1	718.5373	C_39_H_76_NO_8_P/4	10.245	0.12	0.15
7.	PC 32:0e	720.5907	C_40_H_82_NO_7_P	7.923	0.69	1.27
8.	PC 31:0	720.5506	C_39_H_78_NO_8_P	9.700	0.20	0.28
9.	PC 32:2	730.5374	C_40_H_76_NO_8_P	10.409	0.78	0.68
10.	PC 33:1e	732.5879	C_41_H_82_NO_7_P/3	7.898	0.22	0.41
11.	PC 16:0/16:1	732.5615	C_40_H_78_NO_8_P	9.851	3.08	3.16
12.	PC 33:0e	734.6039	C_41_H_84_NO_7_P/2	7.650	0.35	0.21
13.	PC 16:0/16:0	734.5714	C_40_H_80_NO_8_P	9.651	1.46	2.25
14.	PC 34:4e	740.563	C_42_H_78_NO_7_P	8.625	0.17	0.28
15.	PC 34:3e	742.5725	C_42_H_80_NO_7_P	8.365	0.18	0.26
16.	PC 34:2e	744.5921	C_42_H_82_NO_7_P	8.022	0.23	0.31
17.	PC 33:2	744.5538	C_41_H_78_NO_8_P	10.034	0.08	0.15
18.	PC 16:0e/18:1	746.6071	C_42_H_84_NO_7_P	7.637	0.87	1.20
19.	PC 17:0/16:1	746.5754	C_41_H_80_NO_8_P	9.389	0.26	0.46
20.	PC 34:0e	748.6226	C_42_H_86_NO_7_P	7.420	0.41	0.41
21.	PC 33:0	748.5921	C_41_H_82_NO_8_P/3	9.003	0.27	0.18
22.	PC 35:4e	754.5794	C_43_H_80_NO_7_P	8.230	0.08	0.11
23.	PC 34:4	754.5339	C_42_H_76_NO_8_P	10.397	1.62	1.33
24.	PC 34:3	756.5537	C_42_H_78_NO_8_P	10.019	1.66	1.37
25.	PC 16:1/18:1	758.5755	C_42_H_80_NO_8_P	9.515	1.55	1.33
26.	PC 16:0/18:1	760.5882	C_42_H_82_NO_8_P	9.020	3.59	3.37
27.	PC 34:0	762.6052	C_42_H_84_NO_8_P	8.757	0.86	0.55
28.	PC 16:0e/20:5	766.5762	C_44_H_80_NO_7_P	8.282	2.06	2.17
29.	PC 16:0e/20:4	768.5954	C_44_H_82_NO_7_P	7.904	3.60	4.36
30.	PC 16:0e/20:3	770.6110	C_44_H_84_NO_7_P	7.759	0.77	1.05
31.	PC 36:2e	772.6268	C_44_H_80_NO_7_P	7.416	0.50	0.63
32.	PC 35:2	772.5811	C_43_H_82_NO_8_P	9.076	0.20	0.28
33.	PC 36:1e	774.6345	C_44_H_88_NO_7_P	7.076	0.49	0.52
34.	PC 35:1	774.5959	C_43_H_84_NO_8_P	8.695	0.19	0.33
35.	PC 36:6	778.5337	C_44_H_76_NO_8_P	10.421	0.66	0.50
36.	PC 37:5e	780.5907	C_45_H_82_NO_7_P	7.992	0.62	0.53
37.	PC 16:0/20:5	780.5498	C_44_H_78_NO_8_P	9.905	3.46	2.77
38.	PC 17:0e/20:4	782.6112	C_45_H_84_NO_7_P	7.605	0.98	1.01
39.	PC 16:0/20:4	782.5703	C_44_H_80_NO_8_P	9.409	4.76	4.21
40.	PC 16:0/20:3	784.5843	C_44_H_82_NO_8_P	9.175	2.63	2.66
41.	PC 16:0/20:2	786.6031	C_44_H_84_NO_8_P	8.702	3.60	3.42
42.	PC 16:0/20:1	788.6177	C_44_H_86_NO_8_P	8.446	1.12	1.66
43.	PC 38:6e	792.5874	C_46_H_82_NO_7_P	8.067	0.70	0.62
44.	PC 18:0e/20:5	794.6091	C_46_H_84_NO_7_P	7.697	4.26	3.85
45.	PC 18:0e/20:4	796.6243	C_46_H_86_NO_7_P	7.320	7.77	7.18
46.	PC 18:0e/20:3	798.6404	C_46_H_88_NO_7_P	7.009	2.01	2.55
47.	PC 37:3	798.6045	C_45_H_84_NO_8_P/6	8.783	0.17	0.16
48.	PC 18:0e/20:2	800.6484	C_46_H_90_NO_7_P	6.837	1.32	1.37
49.	PC 37:2	800.6126	C_45_H_86_NO_8_P/5	8.352	0.21	0.24
50.	PC 38:7	804.5497	C_46_H_78_NO_8_P	10.103	0.87	0.62
51.	PC 38:6	806.5599	C_46_H_80_NO_8_P	9.579	2.48	1.82
52.	PC 39:5e	808.6155	C_47_H_86_NO_7_P	7.474	0.21	0.21
53.	PC 18:1/20:4	808.5769	C_46_H_82_NO_8_P	9.086	4.00	3.05
54.	PC 39:4e	810.6348	C_47_H_88_NO_7_P	7.047	0.34	0.31
55.	PC 18:0/20:4	810.6025	C_46_H_84_NO_8_P	8.690	3.88	3.60
56.	PC 38:3	812.6167	C_46_H_86_NO_8_P	8.377	1.69	1.55
57.	PC 18:1/20:1	814.6241	C_46_H_88_NO_8_P	8.101	1.54	0.86
58.	PC 40:6e	820.6119	C_48_H_86_NO_7_P	7.522	1.35	1.18
59.	PC 39:6	820.5864	C_47_H_82_NO_8_P	9.069	0.19	0.17
60.	PC 20:1e/20:4	822.6367	C_48_H_88_NO_7_P	7.138	3.11	3.03
61.	PC 39:5	822.5991	C_47_H_84_NO_8_P	8.680	0.39	0.38
62	PC 40:4e	824.6498	C_48_H_90_NO_7_P	6.862	1.98	1.82
63.	PC 39:4	824.6192	C_47_H_86_NO_8_P	8.355	0.21	0.21
64.	PC 40:9	828.5505	C_48_H_78_NO_8_P	10.116	0.84	0.91
65.	PC 40:8	830.5639	C_48_H_80_NO_8_P	9.650	1.28	1.37
66.	PC 40:7	832.5771	C_48_H_82_NO_8_P	9.214	1.66	1.69
67.	PC 20:1/20:5	834.5997	C_48_H_84_NO_8_P	8.722	3.38	3.15
68.	PC 41:5e	836.6457	C_49_H_90_NO_7_P	6.924	0.30	0.42
69.	PC 20:1/20:4	836.6159	C_48_H_86_NO_8_P	8.320	3.74	3.23
70.	PC 20:2/20:2	838.6321	C_48_H_88_NO_8_P	8.023	1.95	1.82
71.	PC 40:3	840.6399	C_48_H_90_NO_8_P	7.753	0.78	0.83
72.	PC 40:5e	850.6595	C_50_H_92_NO_7_P/7	6.748	0.45	0.61
73.	PC 41:5	850.6241	C_49_H_88_NO_8_P	8.121	0.19	0.22

## Data Availability

Study data are available from the corresponding authors upon reasonable request.

## Sample Availability

All materials regarding the sea urchin can be supported by the authors.
